# Modern Thought (Poetical)

**Published:** 1874-05

**Authors:** 


### *MODERN THOUGHT.*

We follow Science in its grim belief
That Time 's a sort of anatomic thief—
A subtle spoiler, who, in open day,
Can steal our flesh and blood and bones away.

And then to pseudo-science we advance,
Discarding Spirit to get rid of Chance;
This leaves but matter and material laws—
The brick and mortar of Effect and Cause !

Behold results ! The body disappears—
Kidnapp'd piece-meal, in every seven years ;
And thus each vagrant transitory elf
Grows up a stranger even to—himself !

Our kith and kin, of high or low estate,
All vanish from us 'ere the age of eight :
Ancestral blood but seven years remains
To run its course through son or daughter's veins.

'Tis true, successive beings seem the same,
And pass for children and assume the name :
But this, we know, is but a fiction bold ;
No real child was ever eight years old.

And brief, alike, the fond parental stage !
The oldest parent is a child in age :
The father dear, and mother, ever blest,
Must change to other people with the rest.

Indeed, not e'en the mystic nuptial tie
Can safely guard the pronouns, You and I :
Two happy hearts, in early life made one,
Get wideiy sever'd ere their race is run.

Protracted wedlock thus becomes, per force,
A round of marriage and of slow divorce :
In vain does constancy intend to cleave ;
Her vows redouble, and—she takes her leave.

And Death is doubtful when he claims a prize ;
He needs must doubt what real person dies :
An inquest only can the point decide,
If still we live or have already died.

L. E. B.

*Atlanta Daily Herald.*]

